# Extensive QTL and association analyses of the QTLMAS2009 Data

**DOI:** 10.1186/1753-6561-4-s1-s11

**Published:** 2010-03-31

**Authors:** Georgia Hadjipavlou, Gib Hemani, Richard Leach, Bruno Louro, Javad Nadaf, Suzanne Rowe, Dirk-Jan de Koning

**Affiliations:** 1 Division of Genetics and Genomics, Roslin Institute and Royal (Dick) School of Veterinary Studies, University of Edinburgh, Roslin, Midlothian, EH25 9PS, UK

## Abstract

**Background:**

We applied a range of genome-wide association (GWA) methods to map quantitative trait loci (QTL) in the simulated dataset provided by the QTLMAS2009 workshop to derive a comprehensive set of results. A Gompertz curve was modelled on the yield data and showed good predictive properties. QTL analyses were done on the raw measurements and on the individual parameters of the Gompertz curve and its predicted growth for each interval. Half-sib and variance component linkage analysis revealed QTL with different modes of inheritance but with low resolution. This was complemented by association studies using single markers or haplotypes, and additive, dominance, parent-of-origin and epistatic QTL effects. All association analyses were done on phenotypes pre-corrected for pedigree effects. These methods detected QTL positions with high concordance to each other and with greater refinement of the linkage signals. Two-locus interaction analysis detected no epistatic pairs of QTL. Overall, using stringent thresholds we identified QTL regions using linkage analyses, corroborated by 6 individual SNPs with significant effects as well as two putatively imprinted SNPs.

**Conclusions:**

We obtained consistent results across a combination of intra- and inter- family based methods using flexible linear models to evaluate a variety of models. The Gompertz curve fitted the data really well, and provided complementary information on the detected QTL. Retrospective comparisons of the results with actual data simulated showed that best results were obtained by including both yield and the parameters from the Gompertz curve despite the data being simulated using a logistic function.

## Background

The QTLMAS2009 data is structured in families and allows both linkage and association approaches to be evaluated. Here we describe a comprehensive set of analyses to detect QTL in the simulated population in order to compare routinely used methods of linkage and association analysis. The half sib method is fast and robust but ignores family information other than parent-offspring relationship being analysed. The variance component analysis is computationally more intensive but models all relationships and is easily extended to non-additive scenarios. Direct association of marker genotypes is computationally fast but requires denser markers and is more susceptible to data stratification. Jointly, these analyses represent a number of models that are expected to give good insight into the genetic architecture of the trait.

## Methods

### Treatment of phenotypic data 

All analyses used the simulated data on 1000 offspring from 20 dams, nested in 5 sire families. Univariate analyses in ASREML [1] were used to estimate heritabilities at each of the time points. The Gompertz growth function, modelling weight over time, was fitted across all trait data using nonlinear regression in SAS. The following parameterization of the Gompertz equation was used: y(t) = Ae^{-e[Be(C-t)/A]}^ , where:

y(t) = yield at time t; A = final yield; B = maximum growth rate; C = age at maximum growth rate.

The Gompertz function was then fitted to trait information for each individual separately and individual estimates of the model parameters A, B, C were extracted. Subsequently, parameter estimates for each individual were employed in the model equation and its derivative to predict yield and growth rate (yield per day), respectively, at the 5 time points for which trait information was available (0 to 530) and at time 600.

### Half-sib QTL analyses

QTL analyses of the simulated phenotypes, the estimated Gompertz parameters A, B, and C and the predicted growth rates at the given time points and at time 600 were conducted using the half-sib QTL analysis, as described by Knott* et al* . [2] and implemented in the web-based software GridQTL [3]. All half- sib analyses were performed for paternal and maternal half-sib families. Empirical thresholds were obtained by permutation tests using 2000 permutations per chromosome. From these chromosome-wide thresholds the following significance levels were derived: chromosome-wide and genome-wide 5% and 1%. QTLs detected at the 1% chromosome-wide significance level were included in the one-QTL model as cofactors. For chromosomes where a single QTL had been identified a two-QTL model was evaluated and the best fitted two-QTL model obtained tested against the best one-QTL model.

### Variance component QTL analyses

A variance component approach was used to look for additive, dominant and imprinted QTL. Following a two-step approach [4], identical-by-descent (IBD) coefficients were estimated for all relationships in the pedigree with the recursive method of Pong-Wong* et al,*[5]. Variance components for each model were estimated using ASReml [1]. The following models were evaluated

(1) **y** =** Xβ** +** Zu** +** e**	(null or polygenic)

(2) **y** =** Xβ** +** Zu** +** Za** + **e**	(additive QTL)

(3) **y** =** Xβ** +** Zu** +** Za** +** Zd** +** e** (additive QTL + dominant QTL)

(4) **y** =** Xβ** +** Zu** +** Z_m_m** +** Z_p_p** +** e** (maternal QTL + paternal QTL)

where** y** is a vector of phenotypic observations,** β** is a vector of fixed effects,** u, a, d, m, p** and** e** are vectors of random additive polygenic effects, additive and dominance QTL effects, maternal and paternal QTL effects and residuals, respectively.** X, Z, W, Zm,** and** Zp** are incidence matrices relating to fixed and random genetic, maternally expressed, and paternally expressed QTL effects, respectively. Variances for polygenic and QTL effects are distributed as follows: var**(u)** =**A**σ ^2^_a_, Var**(a)** =** G**σ^2^_q_, Var**(d)** =** D**σ^2^_d_, Var**(m)** =** G_M_**σ^2^_m_, Var**(p)** =** G_P_**σ^2^_p_, var**(e)** =** I**σ^2^_e_.** A** is the standard additive relationship matrix based on pedigree data only and the relationship matrices. The** G, G_M_, G_P_** and** D** are the appropriate relationship matrices used to model the additive, maternal, paternal and dominant QTL effects at each position tested as outlined by Liu* et al,*[6].

The logarithm of the likelihood ratio test statistic was used to test the presence of a QTL at given locations along the genome. A nominal χ^2^_1_ or χ^2^_2_ was used depending on whether one or two extra parameters were estimated. This has been shown to be conservative as the theoretical distribution is a mixture between 0 and χ^2^_1_ or χ^2^_1_ and χ^2^_2_, respectively[7].

Models (3) vs. (2) were compared to detect dominant effects. Models (4) vs. (1) were compared to test for an additive QTL whilst allowing the maternal and paternal components to vary and (4) vs (2) to test whether the additive effect was better explained by allowing different parental contributions.

### Association studies 

We first tested the level of linkage disequilibrium (LD) using Haploview [8]. For association analyses, we used the simulated phenotypes as well as the Gompertz parameters A, B, C (results not shown). Association analyses were performed using the GRAMMAR approach [10], which comprises two stages. First, ASReml is used to correct each phenotype for polygenic effects; and second, additive, dominant and imprinting models were sequentially fitted against each marker on the residual phenotypic values with an ANOVA test. In the case of a better fit of the imprinting model for a given SNP, we generated a 5% significance level by performing 2000 randomizations where we randomly swapped the maternal and paternal allelic origin for half the offspring. An empirical genome-wide threshold of 5% was generated from 1,000 permutations. We also applied haplotype analyses and exhaustive epistatic searches, but these revealed no additional QTL.

## Results and discussion

### Descriptive statistics

The estimated heritability was ~ 0.50 for all time points varying between 0.46 and 0.50. The heritabilities for the Gompertz parameters A, B and C were 0.45, 0.48 and 0.26, respectively. The LD between adjacent SNP pairs was generally low. Ostensibly, 453 markers spanning a genetic distance of 5 Morgans appeared to be sparse, and the pattern of LD reflects this. Chromosomes 1 to 4 had similar distributions of r^2^ values, the mean between adjacent markers being ~0.15, but chromosome 5 appeared to have much lower LD (Additional file 1).

### Linkage analyses

The results of the half-sib analyses are summarised in additional file 2 and curves for all half-sib analyses are presented in additional file 3. Genome-wide significant QTL were identified for all time points and all chromosomes. A QTL on chromosome 1 (43cM) was highly significant for growth rate and yield across all times from both sire and dam half-sib analyses. On chromosome 2, the sire analysis identified 3 significant QTL while the dam analysis revealed two QTL (Additional file 2) On chromosome 3, two QTL were detected from the sire half-sib analysis while the dam analysis showed evidence for two QTL (Additional file 2) The sire half-sib regression resulted in one QTL on chromosome 4 (78-79cM) which was significant at early times. On chromosome 4, the dam analysis identified two QTL at 5-8cM and 86cM for yield times 0-132. Finally, from the sire analysis a QTL on chromosome 5 was significant at 75-76cM for growth rate from time 132 onward and yield for time points 265-530, whereas the dam regression revealed a QTL (99cM) for yield and growth rate at early times. Major QTL on chromosome 1 (38-44 cM) from both parental analyses and paternally inherited ones on chromosomes 2 and 5 were detected for the Gompertz parameters for final yield (A) and maximum growth rate (B).

The variance component analyses are summarized in Table 1, and depicted in additional file 4. Because the VC analyses model QTL from both parents simultaneously, the results reflect a mix between the two half-sib analyses. The VC analyses confirm the highly significant QTL on chromosome 1, explaining around 35% of the variance and acting additively. The parent-of-origin specific models that allow for imprinting show potential imprinting effect for chromosomes 2, 3, and 4 (Table 1). These are in line with the observed differences for the alternative half-sib models. There is also indication of dominant QTL on chromosomes 4 and 5 at 75 and 30cM, respectively, affecting time 397.

**Table 1:** Summary of most significant QTL results for yield from variance component analyses.

		QTL		Time	% variance
Model	Chromosome	Position	LRT	Point	explained
		(cM)^1^			by QTL
Additive	1	43	135.6**	530	35
Additive	2	5	25.42**	530	6.4
Additive	2	38	24.1**	397	7.3
Additive	3	17	14.05**	0	5.03
Additive	3	93	8.6**	265	4.92
Additive	4	37	15.61**	0	5.66
Additive	4	77	27.85**	0	7.16
Additive	5	73	11.98**	530	5.0
Dominant^2^	4	75	7.32**	397	2.3/5.5
Dominant^2^	5	30	2.44	397	0/3.4
Imprinting^3^	2	9	4.5*	132	0/5.5
Imprinting^3^	2	62	3.4	530	0/4.6
Imprinting^3^	3	76	3.0	265	2.6/0
Imprinting^3^	3	76	3.6	397	2.4/0
Imprinting^3^	4	9	8.3**	0	6.2/0

^1^QTL position is defined relative to the first marker (SNP) present in the genetic map for each chromosome; first marker positioned at 1 cM.

^2^For dominant QTL, the LRT is the test of model 3) against model 2). The variance explained by the QTL is given as additive /dominance

^3^ For imprinted QTL, the LRT is the test of model 4) against model 2). The variance explained by the QTL is given as maternal /paternal

*/** Significance threshold P < 0.05/0.01 assuming the null test statistic follows a χ^2^1 distribution

### Association analyses using GRAMMAR 

**Figure 1:**
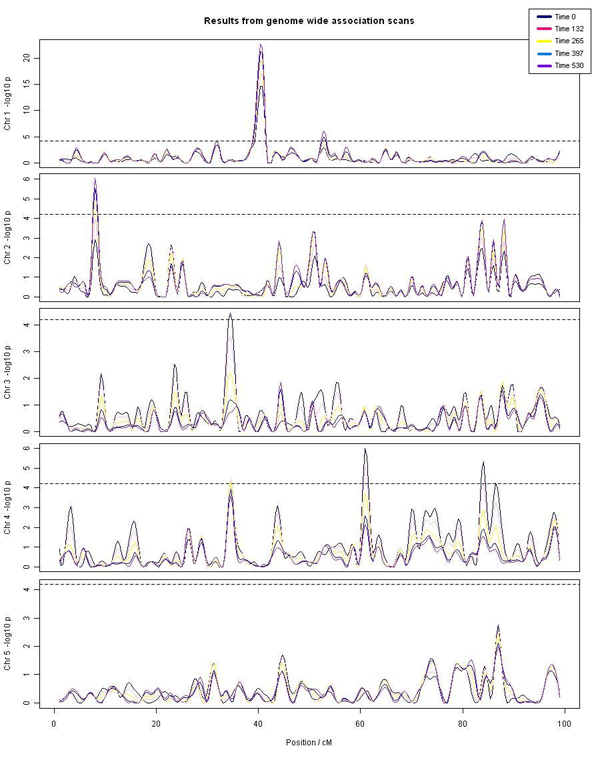
Profile of association across all chromosomes over time

Figure 1 shows the results of fitting an additive model to all individual SNPs over times. This revealed six genome-wide significant SNPs affecting the five yield traits (Table 2, Additional file 5). The major SNP effect was at 44.5 cM (SNP 37) on chromosome 1, explaining up to 10% of phenotypic variation in yield. Most other significant SNPs had much more modest effects, explaining between 1 and 3 % of phenotypic variation. A parent-of-origin specific model revealed two additional genome-wide significant QTL. The first one (SNP 53 at 56.6 cM) was paternally expressed. For this SNP, the empirical p value was 0.03. Two out of the 5 sires had both alleles for the SNP. The contrast effect of Aa vs. aA was assessed across the 2 sires and was significant (p<0001, N=194) with the most important effect on late growth. Another parent-of origin QTL, which was expressed paternally, was detected on chromosome 2 (SNP 138 at 48.5 cM). For the SNP 138, the empirical p value was about 0.0005. Only one of the 5 sires had both alleles. The within family contrast effect was significant (p=001, N=93) with the most important effect on early growth. The different analysis models for selected SNPs are compared in additional file 6.

**Table 2:** Most significant associations with single SNPs.

		SNP number		Time Point^a^	% variance
Model	Chromosome	(cM)	-log_10_P		explained by QTL^a^
Additive	1	37 (44.5)	15.0-22.6	0-530	6.7-9.9
Additive	2	98 (3.6)	4.0-6.1	132-530	1.8-2.8
Additive	2	174 (88.3)	3.3-4.6	132-530	1.3-1.6
Additive	3	222 (31.1)	3.0-5.2	0-265	0.9-1.77
Additive	4	338 (71.7)	3.0-7.3	0-530	1.0-2.8
Dominant	4	315 (38.8)	4.4-5.1	0-530	1.7-2.0
Imprinting	1	53 (55.6)	3.9	530	1.6
Imprinting	2	138 (48.5)	3.6	0	1.5

^a^ For SNPs with nominal P < 0.001

### Overall comparison 

The linkage analyses give a single QTL on chromosome 1, up to 3 QTL regions on chromosomes 2, 3, and 5 and 2 QTL regions on chromosome 4 (Additional file 1 and Table 1). There are some slightly more speculative QTL with potential imprinting effects on chromosomes 2, 3, and 4 but these require further scrutiny. The association study shows convincing evidence for 6 SNPs, that all coincide with QTL regions. There are two putatively imprinted SNPs, but these show little concordance with the putative imprinted regions from the VC Analyses.

## Epilogue

The retrospective comparison of the performance of the methods used here with the simulated data is shown in additional file 7. Because the Gompertz growth curve fitted the yields well, half-sib regression analysis of the Gompertz model descriptors performed better than all other methods employed, even though the actual QTL were simulated for the 3 parameters of the logistic function. The half-sib, association and VC analyses of yield data detected 12, 6 and 10 out of the 18 simulated QTL respectively with 1, 2 and 5 false positives. Analysis of the growth model descriptors (growth rate and the Gompertz model parameters) resulted in 15 QTL with no false positives. The association analysis tended to underestimate the variance explained by the QTL with the VC analysis giving the most accurate estimation of variance explained (S2). Comparison of the methods clearly demonstrates the risks of false detection of non-additive segregation. The imprinted QTL falsely detected by the VC and association analyses can be explained by segregation of QTL, where only a limited number of parents are heterozygous for the QTL. As a result, the QTL effect may appear to come from the parents of a single sex only. The apparent parental origin differences are clearly illustrated by the differences in half-sib regression results (Additional files 2 and 3). The dominant effects, however, are more difficult to interpret, perhaps coming from higher yield within a particular full sib family thus masquerading as dominance. Results from these extended models provide a valuable insight and perhaps serve as a warning on the effects of data structure on results from non additive models.

## List of abbreviations used

QTL: Quantitative Trait Locus; LD: Linkage Disequilibrium; GRAMMAR: Genome- wide Rapid Analysis using Mixed Models And Regression

## Competing interests

The authors declare that they have no competing interests.

## Authors' contributions

GH1, GH2, RL, BL, JN, and SR carried out the analyses and contributed parts of the manuscript. DJK coordinated the analyses and drafted the overall manuscript. All authors have read and contributed to the final text of the manuscript.

## Supplementary Material

Additional file 1

Additional file 2

Additional file 3

Additional file 4

Additional file 5

Additional file 6

Additional file 7
