# Supplementary material for: Extensive QTL and association analyses of the QTLMAS2009 Data
Source: BMC Proc. 2010 Mar 31;4(Suppl 1):S11. doi: 10.1186/1753-6561-4-s1-s11 (PMC2857842; doi:10.1186/1753-6561-4-s1-s11)
Supplement: Additional file 1 [file 1753-6561-4-S1-S11-S1.pdf]

Analysis of LD structure for each chromosome

Chromosome	Mean r^2 between adjacent markers	Mean maximum pair-wise r^2 for each marker
1	0.146	0.506
2	0.154	0.582
3	0.159	0.523
4	0.124	0.496
5	0.082	0.387
