# Supplementary material for: Extensive QTL and association analyses of the QTLMAS2009 Data
Source: BMC Proc. 2010 Mar 31;4(Suppl 1):S11. doi: 10.1186/1753-6561-4-s1-s11 (PMC2857842; doi:10.1186/1753-6561-4-s1-s11)
Supplement: Additional file 2 [file 1753-6561-4-S1-S11-S2.pdf]

Summary of QTL results from half-sib analyses.

Trait	Chromosome	QTL Position (cM)[1]	F-ratio[2]	Time Range of Significance[3]	% Vp Explained by QTL[4]
**Paternal half-sib analyses**					
Yield	1	41-43	8.01-10.87	0-530	21
Gompertz Growth rate	1	40-44	7.75-17.13	0-600	30
Gompertz Parameters	1	38-40	14.64-22.14	A, B, C	38, 28, 26
Yield	2	5-6	3.31-6.2	0-530	12
Yield	2	91	4.24-4.69	0-530	9
Gompertz Growth rate	2	2-6	7.88-7.95	397-600	13
Gompertz Growth rate	2	49	7.37-8.47	132-265	15
Gompertz Growth rate	2	91	4.83	0	4
Gompertz Parameters	2	1-2	7.53-8.0	A, B	13, 14
Yield	3	27	4.65	0	9
Gompertz Growth rate	3	27	4.97	0	4
Gompertz Growth rate	3	99	3.00	265	4
Yield	4	78-79	4.13-7.57	0-397	13
Gompertz Growth rate	4	79	3.27-7.27	0-265	10
Yield	5	76	4.22-4.52	397-530	8
Gompertz Growth rate	5	75-76	3.66-4.86	132-600	8
Gompertz Parameters	5	74-75	4.1-4.87	A,B	6, 8
**Maternal half-sib analyses**					
Yield	1	44-45	6.01-7.48	0-530	47
Gompertz Growth rate	1	43-45	5.89-7.38	0-600	46
Gompertz Parameters	1	43-44	6.34-7.15	A, B	39, 45
Yield	2	39	2.13-2.99	0-530	16
Gompertz Growth rate	2	2	1.98-2.05	530-600	8
Gompertz Growth rate	2	37	2.16	0	11
Gompertz Parameters	2	2	2.02-2.09	A, B	9, 8
Yield	3	78-79	2.32-2.44	0-530	11
Gompertz Growth rate	3	78-79	2.08-2.31	0-397	11
Gompertz	3	87	2.02-2.04	530-600	8

Growth rate					
Gompertz Parameters	3	78	2.06	A	8
Gompertz Parameters	3	87	2.04	B	8
Yield	4	5-8	2.34-3.08	0-132	18
Yield	4	38	2.01	265	8
Yield	4	86	2.08-2.57	0-132	13
Gompertz Growth rate	4	3	2.59	0	11
Gompertz Parameters	4	82	1.91	C	7
Yield	5	99	1.97-2.25	0-265	10
Gompertz Growth rate	5	99	2.24	0	11

[1]QTL position is defined relative to the first marker (SNP) present in the genetic map for each chromosome; first marker positioned at 1 cM.

[2]F-statistics given as range across time points for which the QTL was deemed significant chromosome-wise with at least P-values < 0.05 (determined by permutation testing).

[3]Refers to at least chromosome-wide significance for all traits

[4]Vp refers to the phenotypic variance for each trait. The proportion of Vp due to the QTL was estimated as $4x(1-RMSQ_{full}/RMSQ_{reduced})$, where RMSQ is the residual mean square estimate for each model and “full” is the model with the QTL effect fitted and “reduced” is the model without the QTL effect (Knott *et al.* 1996). For each QTL, it was estimated at the time point when the significance was highest.
