# Supplementary material for: Extensive QTL and association analyses of the QTLMAS2009 Data
Source: BMC Proc. 2010 Mar 31;4(Suppl 1):S11. doi: 10.1186/1753-6561-4-s1-s11 (PMC2857842; doi:10.1186/1753-6561-4-s1-s11)
Supplement: Additional file 3 [file 1753-6561-4-S1-S11-S3.pdf]

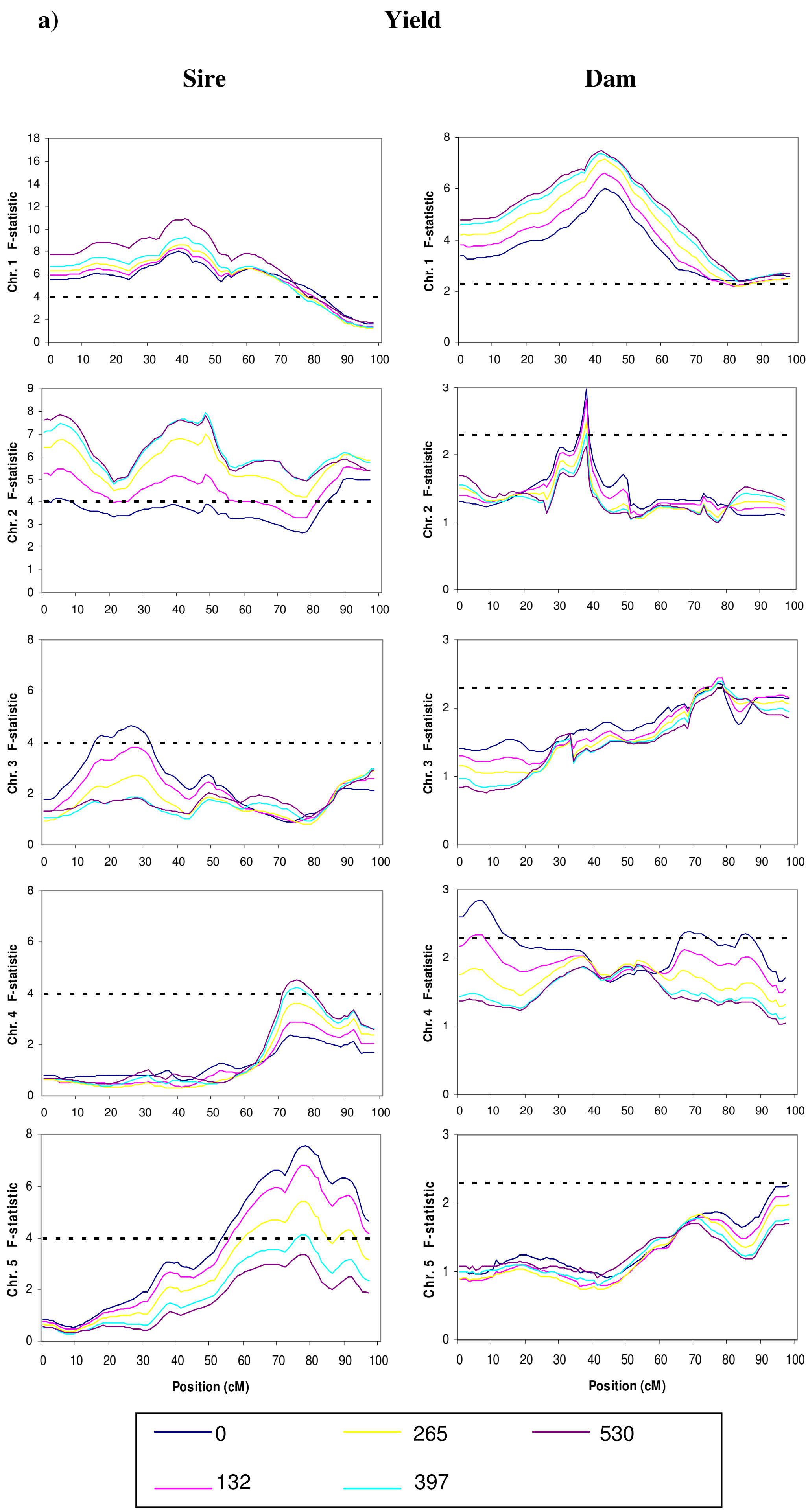
a)
Yield
Sire
Dam
Chr. 1 F-statistic
Chr. 2 F-statistic
Chr. 3 F-statistic
Chr. 4 F-statistic
Chr. 5 F-statistic
Position (cM)
Position (cM)
0
265
530
132
397

b) **Growth Rate**

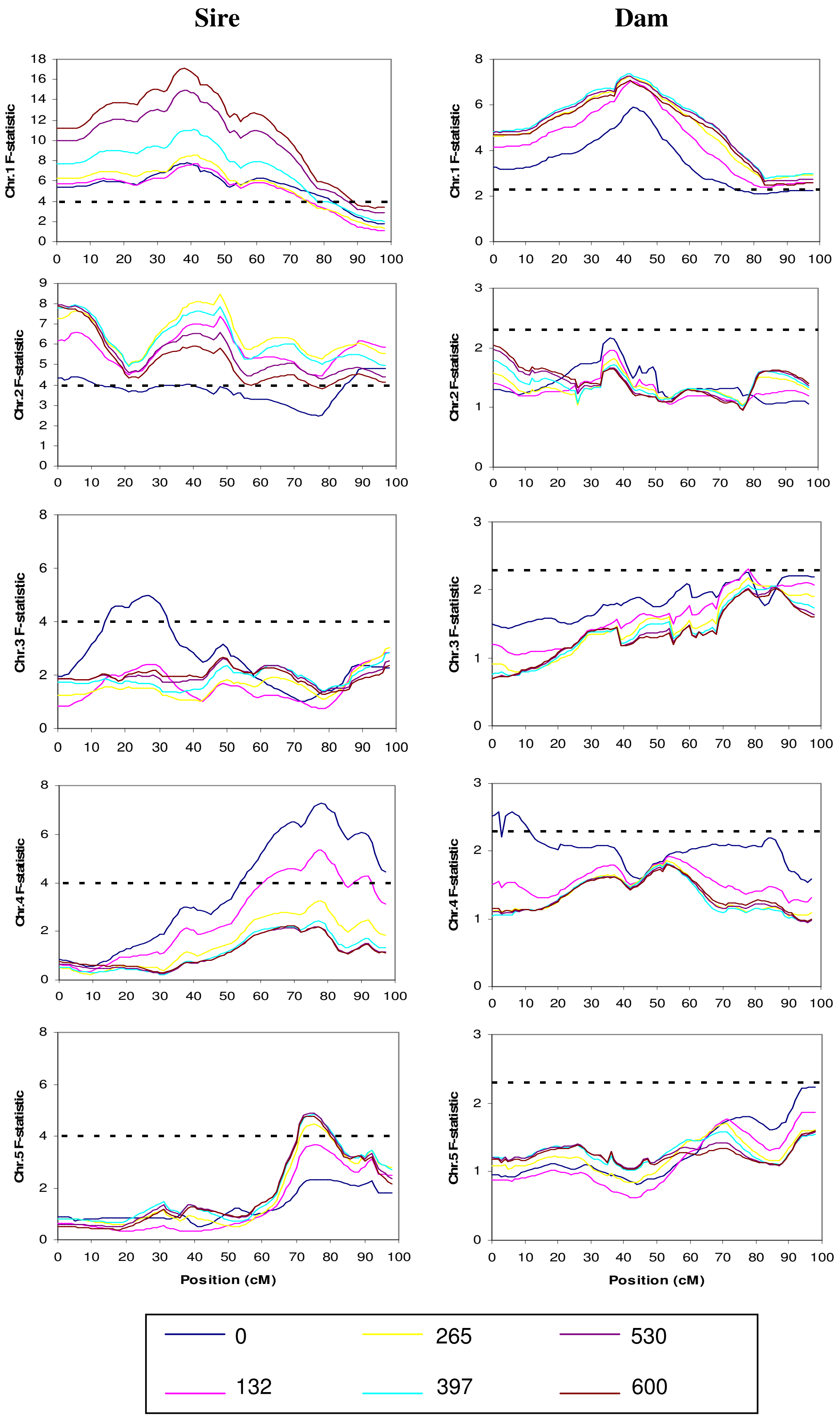

Figure S1. QTL curves for paternal (left) and maternal (right) half-sib analyses.

a) Analyses of yield at the five given time points (0, 132, 265, 397, 530).

b) Analyses of growth rate predicted using the Gompertz model at the five given time points and at time 600.

Genome-wide significance thresholds ($P<0.05$) were determined via permutation testing for each half-sib analysis. The approximate value of the threshold was 4 for the sire analyses and 2.3 for the dam analyses (threshold shown with a dashed line in the diagrams).
