# Supplementary material for: Extensive QTL and association analyses of the QTLMAS2009 Data
Source: BMC Proc. 2010 Mar 31;4(Suppl 1):S11. doi: 10.1186/1753-6561-4-s1-s11 (PMC2857842; doi:10.1186/1753-6561-4-s1-s11)
Supplement: Additional file 4 [file 1753-6561-4-S1-S11-S4.pdf]

Chromosome 1 Additive model

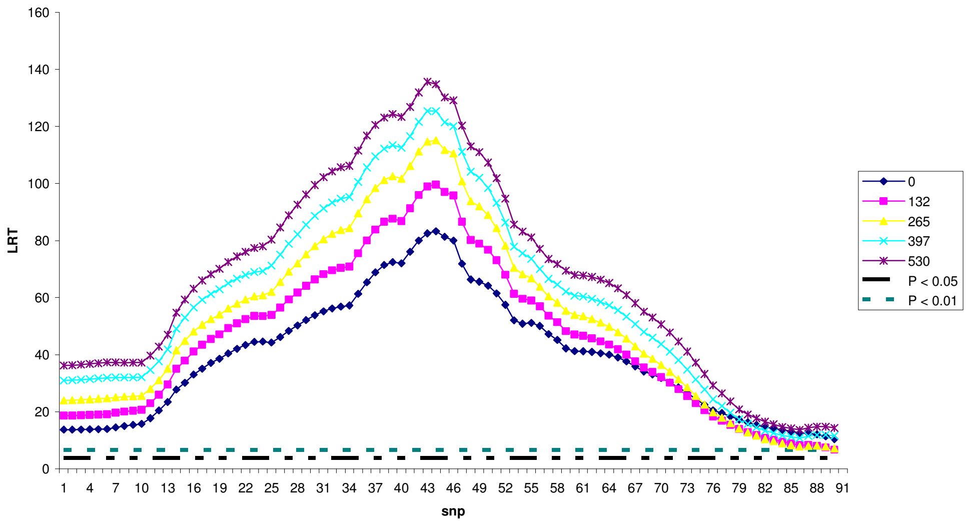

Chromosome 2 Additive model

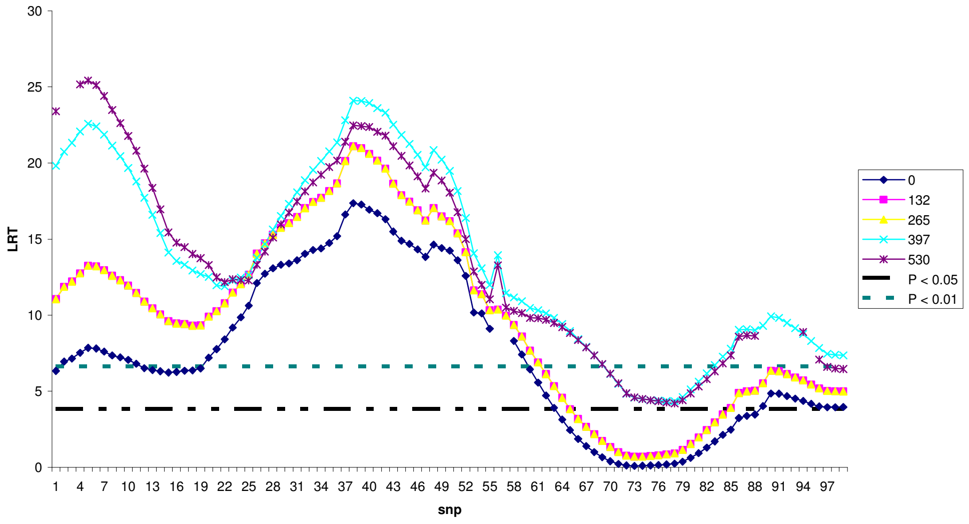

Chromosome 3 Additive model

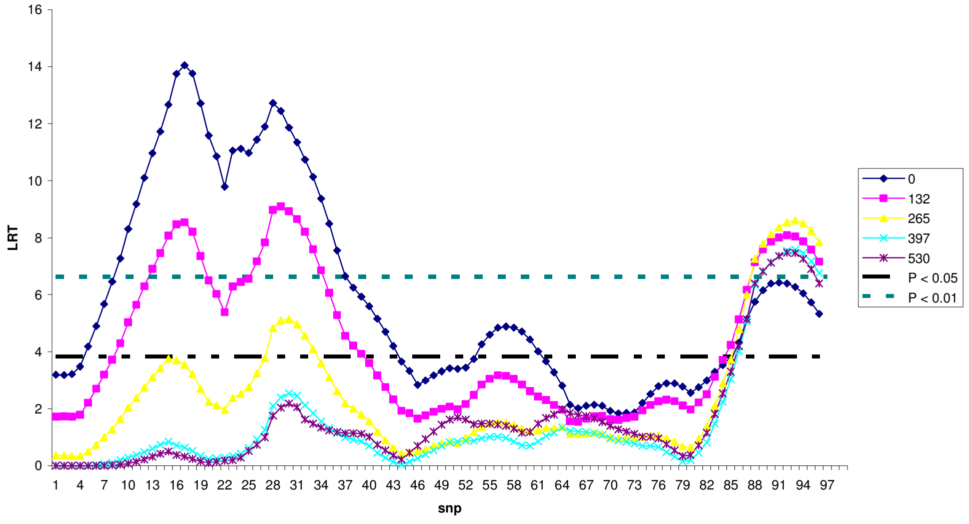

Chromosome 4 Additive model

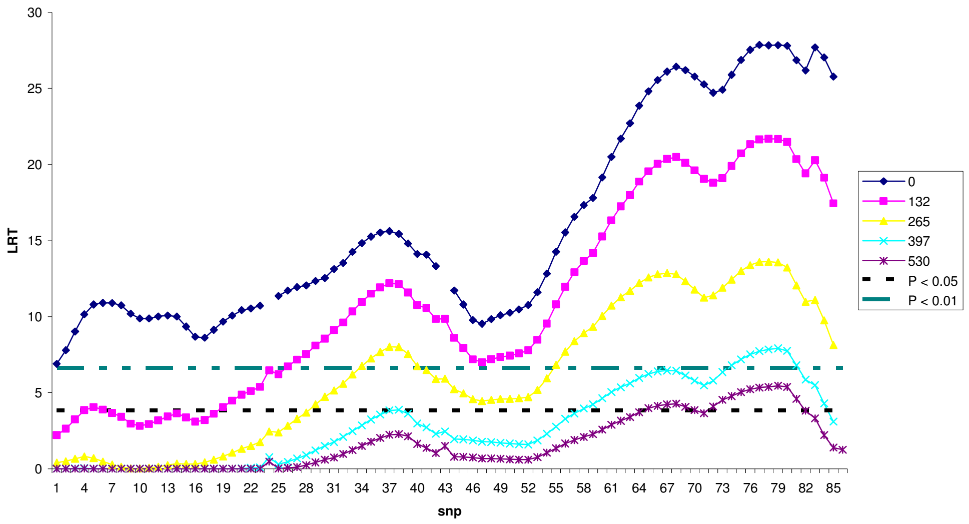

Chromosome 5 Additive model

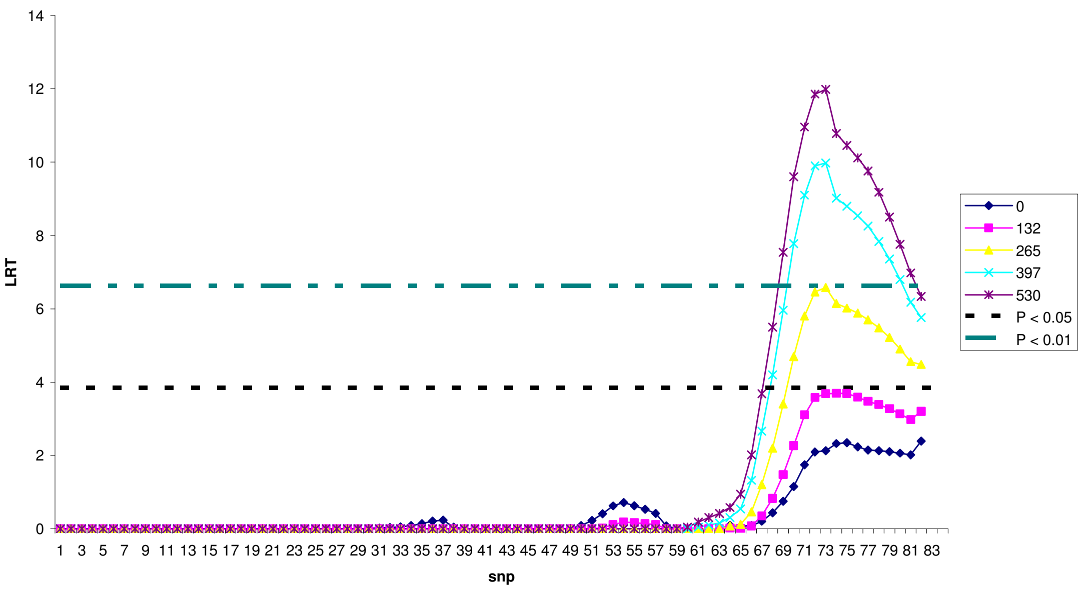

Chromosome 4 Dominance model

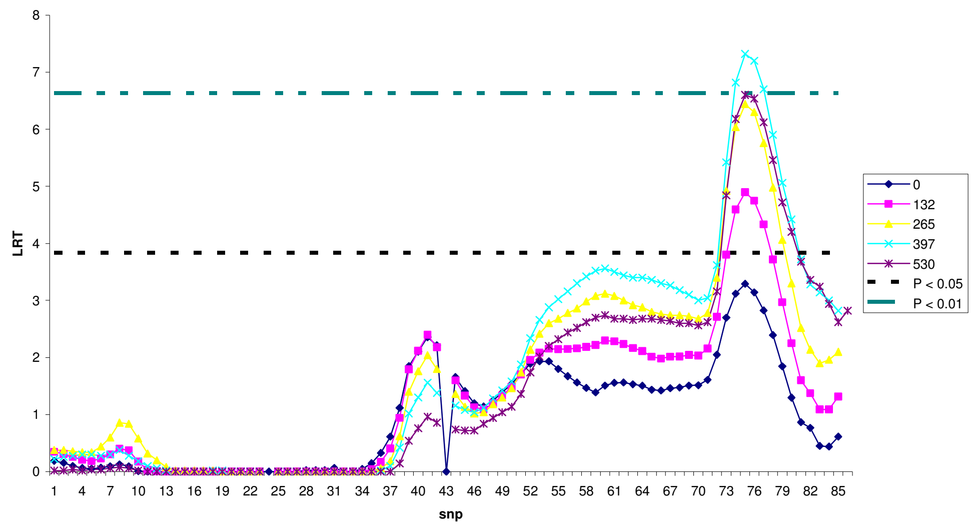

Chromosome 5 Dominance Model

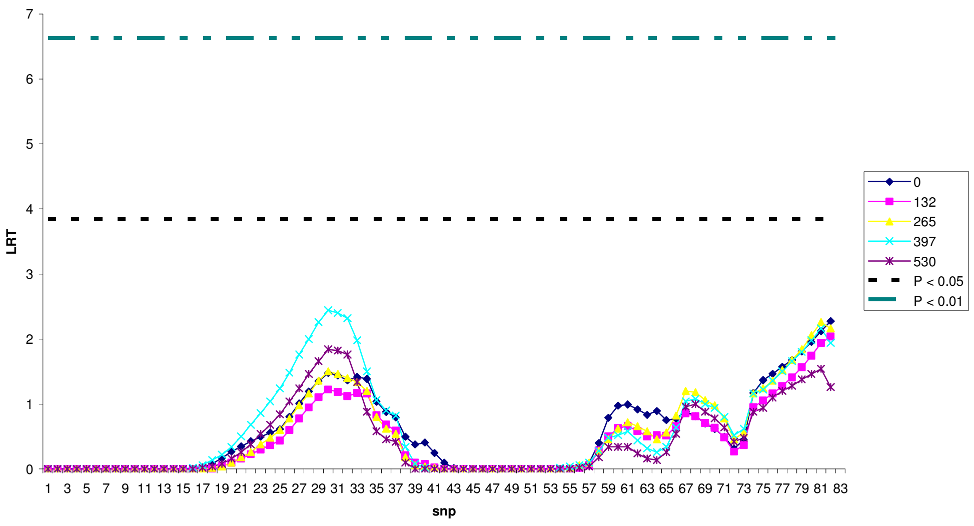

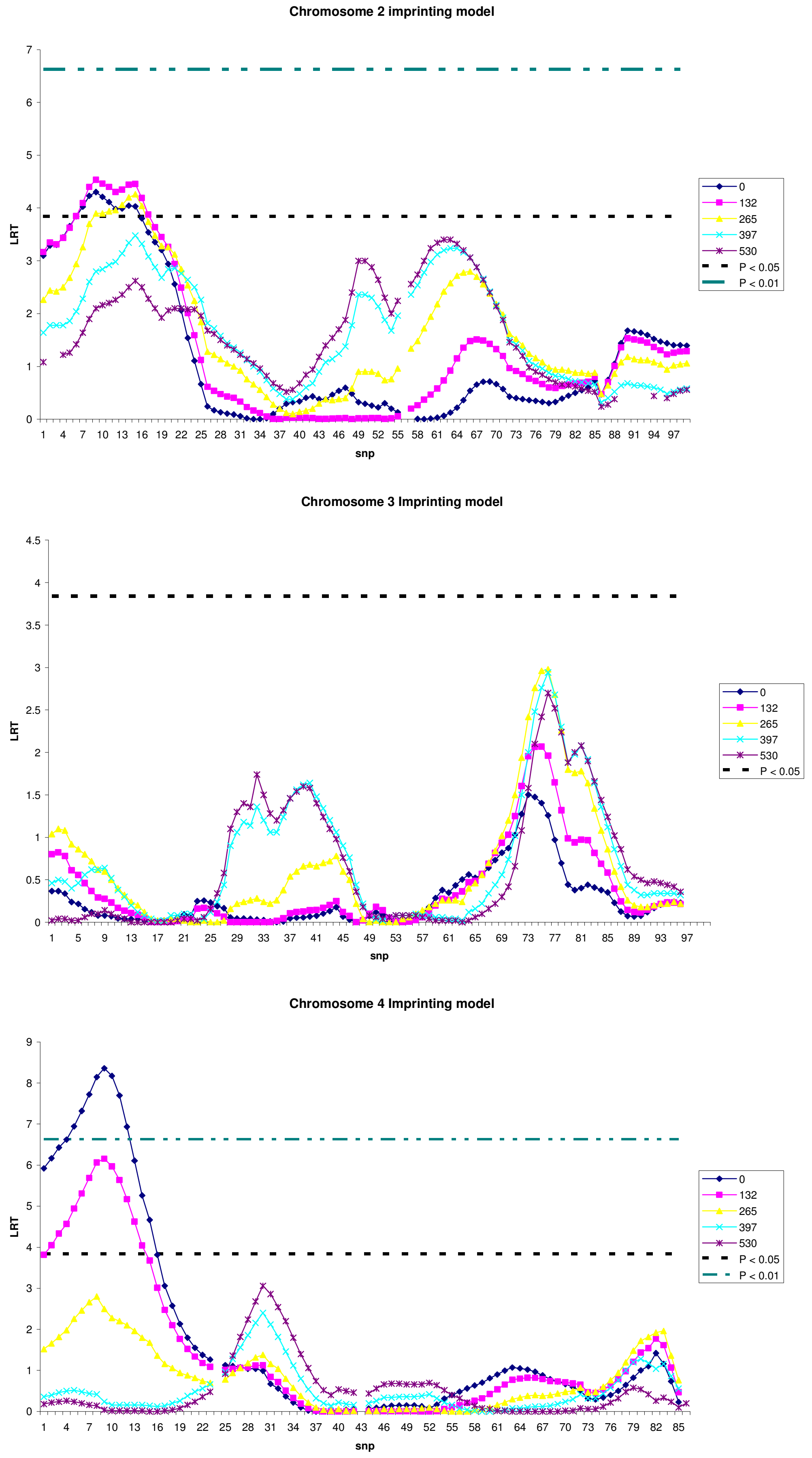

Figure S2. QTL curves for VC analyses under additive (page 1,2), dominant (p3) and imprinting (p4) models. All test statistics are against the H0 of no QTL.
