# Supplementary figures and images for: Extensive QTL and association analyses of the QTLMAS2009 Data
Source: BMC Proc. 2010 Mar 31;4(Suppl 1):S11. doi: 10.1186/1753-6561-4-s1-s11 (PMC2857842; doi:10.1186/1753-6561-4-s1-s11)

**Chr 1, Marker 37**

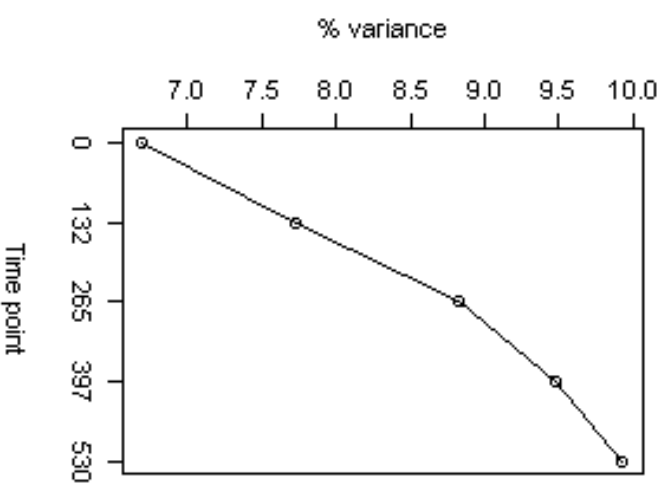

**Chr 2, Marker 8**

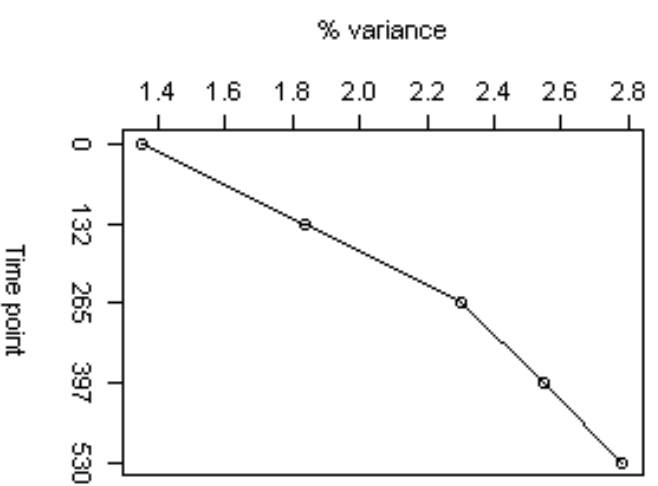

**Chr 2, Marker 84**

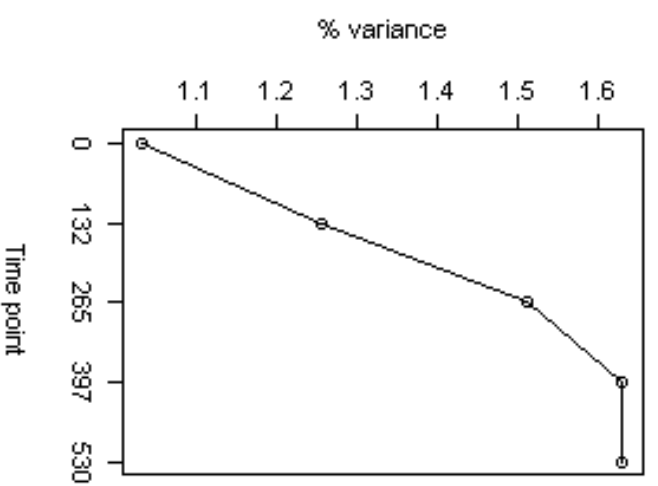

**Chr 3, Marker 33**

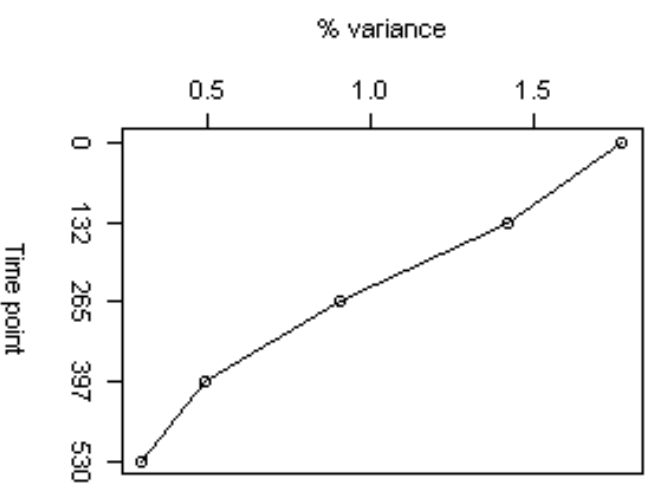

**Chr 4, Marker 30**

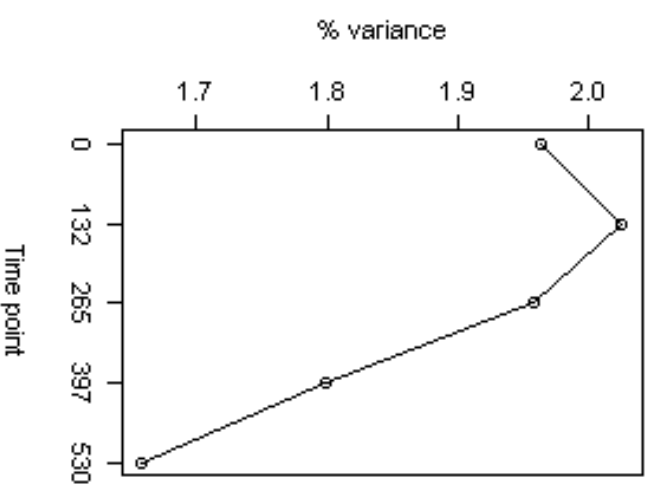

**Chr 4, Marker 53**

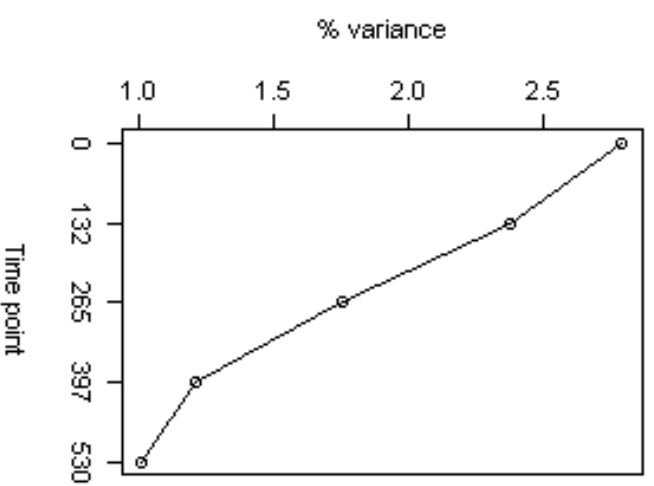

Supplement: Additional file 5 [file 1753-6561-4-S1-S11-S5.pdf]
