# Supplementary material for: Extensive QTL and association analyses of the QTLMAS2009 Data
Source: BMC Proc. 2010 Mar 31;4(Suppl 1):S11. doi: 10.1186/1753-6561-4-s1-s11 (PMC2857842; doi:10.1186/1753-6561-4-s1-s11)
Supplement: Additional file 6 [file 1753-6561-4-S1-S11-S6.pdf]

Comparison of models describing QTL effects from association analysis

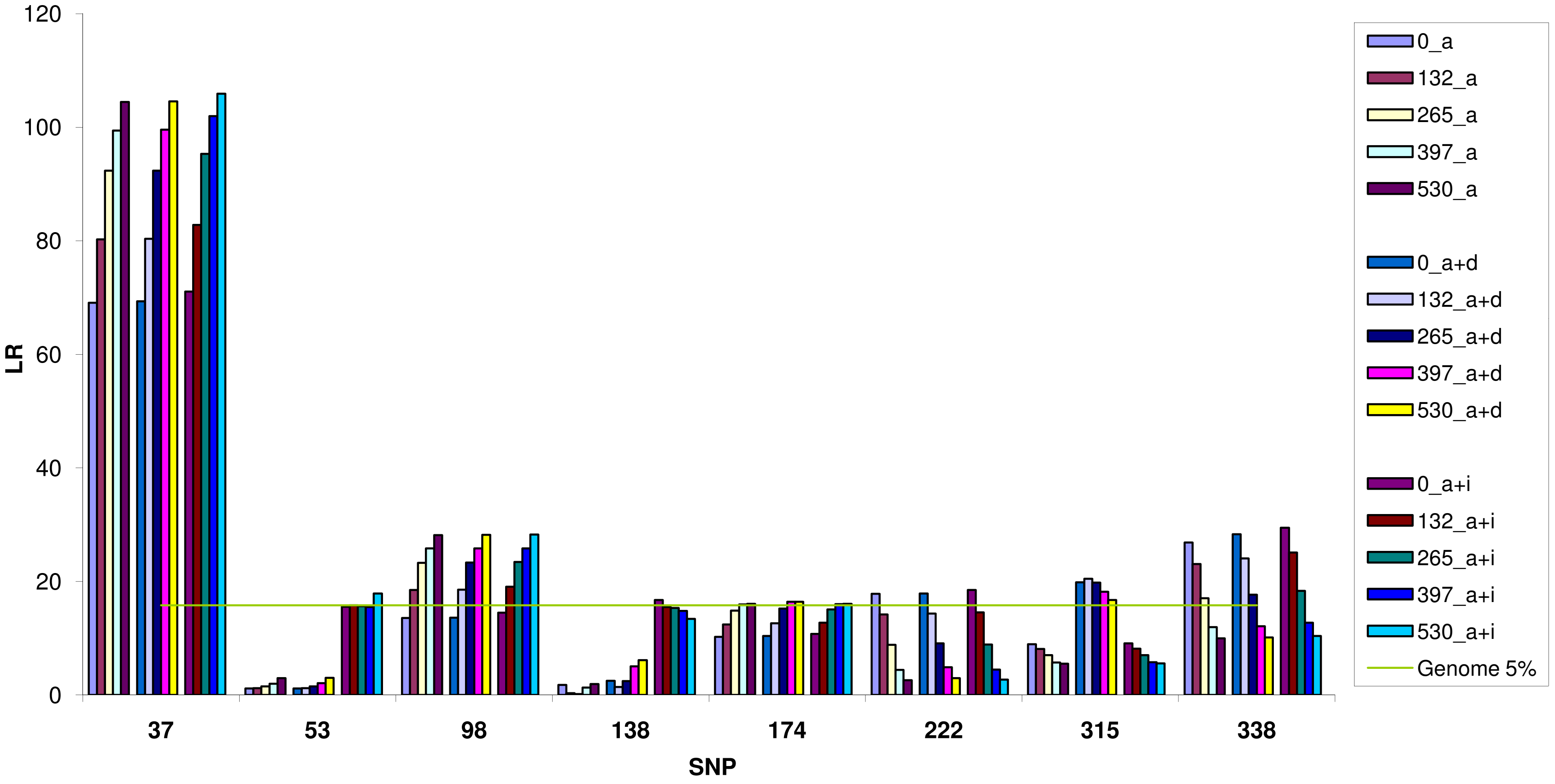

The results on 5 different time points (left to right: time 0 to time 530) with three different models (left to right: a, a+d or a+i; where, a =additive, d= dominance and i=imprint
