# Supplementary material for: Extensive QTL and association analyses of the QTLMAS2009 Data
Source: BMC Proc. 2010 Mar 31;4(Suppl 1):S11. doi: 10.1186/1753-6561-4-s1-s11 (PMC2857842; doi:10.1186/1753-6561-4-s1-s11)
Supplement: Additional file 7 [file 1753-6561-4-S1-S11-S7.pdf]

Performance of different analyses in estimating the simulated QTL for the QTL-MAS workshop 2009.

Data Simulated					Estimated QTL variance component											
					Sire Half-sib Regression Analysis [1]					Dam Half-sib Regression Analysis [1]						
Chr.	Parameter[3]	Actual QTL Position (cM)	Actual QTL Var (%Vp)	No. analyses sig	Yield	Growth Rate	Gompertz Parameters			Yield	Growth Rate	Gompertz Parameters			Variance Component analysis	Association Analysis [2]
							A	B	C			A	B	C		
1	A	42	29.3	11	**21	**29.9	**38	**28	**26	**47	**46	**39	**45		** 35	**19.4
1	IP	54	32.3	1					♦·3.1							
1	GR	88	23.4	1	♦·5.0											
2	A	5	4.55	10	**12.0	**13.4	**12.7	**13.6	*4.9		*8.4	* 8.0	* 8.0		** 6.4	**5.4
2	IP	33	33.02	6					*4.3	**16	*11.4	♦·6.0	♦·6.0		** 7.3	
2	GR	49	48.89	1		**14.6										
2	A	89	3.7	6	**9.0	**3.9	♦·3.9	*4.5		♦·5						**2.8
3	IP	7	3.5	0												
3	GR	26	4.7	6	**9.0	**3.9	♦·3.2		♦·3.0						5.03	**3.2
3	IP	56	3.8	2		♦·3.3		♦·3.3								
3	A	90	4.1	7	♦·5.0	*4.0				**·11	*8.3	*·8.0	*8.0		** 4.92	
4	GR	10	5.9	3						**18	**11.4				** 6.2 IMP	
4	IP	37	3.2	4					*4.7	*8					** 5.66	**3.6
4	A	70	3.3	3			♦·3.9								** 7.16	**5.2
4	GR	86	6.6	4	**·13.0	**9.8				**13				*7.3		
5	GR	31	4.6	2									♦·5.0		3.4 DOM	
5	IP	60	3.7	3	♦·5.0		♦·3.4	♦·3.5								
5	A	77	2.5	6	**8.0	**7.5	**6.1	**7.6			♦·5.7				** 5.0	

[1]** refers to genome-wide significant QTL, whereas * corresponds to chromosome-wide significant QTL. Nominally significant at P<0.05= ♦, and ▪ if the they are also +/-10cM.

[2]All identified QTL (SNPs) were within ±5cM and a significant association with yield was identified (nominal P<0.001).

[3]Parameter from logistic function used to simulate data asymptote (A), relative growth rate (GR) and inflection point (IP)
